# Supplementary material for: Cultural differences in social support seeking: The mediating role of empathic concern
Source: PLoS One. 2021 Dec 30;16(12):e0262001. doi: 10.1371/journal.pone.0262001 (PMC8718000; doi:10.1371/journal.pone.0262001)
Supplement: S1 Appendix — (PDF) [file pone.0262001.s001.pdf]

S1 Appendix. Scales used in this research.

Empathic concern (Davis, 1980)

English version.

	1: Does Not Describe Me Well	2	3	4	5: Describe Me Very Well
I often have tender concerned feelings for people less fortunate than me.	1	2	3	4	5
Sometimes I don't feel very sorry for other people when they are having problems.	1	2	3	4	5
When I see someone being taken advantage of, I feel kind of protective towards them.	1	2	3	4	5
Other people's misfortune do not usually disturb me a great deal.	1	2	3	4	5
When I see someone being treated unfairly, I sometimes don't feel very much pity for them	1	2	3	4	5
I am often quite touched by things that I see happen	1	2	3	4	5
I would describe myself as a pretty soft-hearted person.	1	2	3	4	5

Japanese version.

	1: 全くあてはまらない	2	3	4	5: 非常によく当てはまる
自分より不運な人たちを心配し、気にかけることが多い。	1	2	3	4	5
他の人たちが困っているのを見て、気の毒に思わないことがある。	1	2	3	4	5
誰かがいいように利用されているのをみると、その人を守ってあげたいような気持ちに なる。	1	2	3	4	5
他の人たちが不運な目にあっているのは、たいてい、それほど気にならない。	1	2	3	4	5
誰かが不公平な扱いをされているのをみたときに、そんなにかわいそうだと思わないことがある。	1	2	3	4	5
自分が見聞きした出来事に、心を強く動かされることが多い。	1	2	3	4	5
自分は思いやりの気持ちが強い人だと思う。	1	2	3	4	5

Social support seeking (Carver, 1997)

English version:

	1: Not at all	2	3	4	5: Very much
I try to get emotional support from others.	1	2	3	4	5
I try to get advice or help from other people about what to do.	1	2	3	4	5
I get comfort and understanding from someone.	1	2	3	4	5
I get help and advice from other people.	1	2	3	4	5

Japanese version.

	1: 全くあてはまらない	2	3	4	5: よくあてはまる
他者から感情的なサポート（支え）を得ようとする。	1	2	3	4	5
何をするべきか，他者からアドバイスを得ようとする。	1	2	3	4	5
他者からの慰めや理解を得ようとする。	1	2	3	4	5
他者からの助けやアドバイスを得ようとする。	1	2	3	4	5

Relational concern.

English version.

	1: Not at all	2	3	4	5: Very much
I'm concerned that if I tell the people I am close to about my problems, they would be hurt or worried for me.	1	2	3	4	5
If something were bothering me, I would not want to disrupt my social group by sharing it.	1	2	3	4	5
I wouldn't seek help because I think that others who are close to me will take care of my needs without me having to ask.	1	2	3	4	5
I can save face by solving my problems myself.	1	2	3	4	5
If I discuss my problems with the people I am close to, it makes it a bigger problem than if I keep it to myself.	1	2	3	4	5
I would rather not tell the people I am close to my problems because they would blow them out of proportion.	1	2	3	4	5
I would not need to ask for help because others will probably offer help without me asking.	1	2	3	4	5
To preserve the happiness of my peer group, I try to keep my problems to myself.	1	2	3	4	5
The people I am close to would be ashamed if I made my problems known to others.	1	2	3	4	5
I don't want to ask for support for my problems because people might judge me negatively because of my problems.	1	2	3	4	5
I would be embarrassed to share my problems with the people I am close to.	1	2	3	4	5
I wouldn't want to make the people I am close to feel stressed about my problems.	1	2	3	4	5

I would rather keep my problems to myself than risk criticism from the people I am close to.	1	2	3	4	5

Japanese version.

	1: 全く重要ではない	2	3	4	5: とても重要である
自分が抱えている問題を親しい人々に話すと、そのことでその人たちが傷ついたり、私のことを心配してくれたりすることが気になる。	1	2	3	4	5
私が困っていたとしても、それをみんなに知ってもらうことで所属しているグループの輪を乱すことはしたくない。	1	2	3	4	5
私の親しい人たちは、何も言わなくても私に何が必要か気にかけてくれるので、こちらからそういった助けを求めることはしない。	1	2	3	4	5
私は自分で自分の問題を解決することでメンツを保てる。	1	2	3	4	5
親しい人たちにその問題を打ち明けてしまうと、それを誰にも言わない場合と比べ、問題が大きくなってしまう。	1	2	3	4	5
親しい人たちに自分の問題を話すと、彼らがそれを大げさに騒ぎ立てるので、彼らには問題を打ち明けたくない。	1	2	3	4	5
周囲の人たちは、たぶんこちらから頼まなくても助けてくれるので、私の方からそういった助けを求めなくてもいい。	1	2	3	4	5

仲間のグループ内の幸せを維持するために、自分の問題は自分の中に留めておくようにする。	1	2	3	4	5
もし私が親しい人たちに自分の問題を知らせたとしたら、彼らは恥ずかしい思いをするだろう。	1	2	3	4	5
私の問題のせいで、人は私のことを悪く判断するかもしれないので、助けを求めることはしたくない。	1	2	3	4	5
親しい人たちに自分の問題を打ち明けるのが恥ずかしい。	1	2	3	4	5
私の問題のせいで、親しい人たちにストレスを与えたくない。	1	2	3	4	5
親しい人たちから批判される危険を負うのだったら、むしろその問題については自分の中で留めておくほうがいい。	1	2	3	4	5

Loneliness (Russell et al., 1980).

English version.

	1: Never	2: Rarely	3: Sometimes	4: Often
I feel in tune with the people around me.	1	2	3	4
I lack companionship.	1	2	3	4
There is no one I can turn to.	1	2	3	4
I do not feel alone.	1	2	3	4
I feel part of a group of friends.	1	2	3	4
I have a lot in common with the people around me.	1	2	3	4
I am no longer close to anyone.	1	2	3	4
My interests and ideas are not shared by those around me.	1	2	3	4
I am an outgoing person.	1	2	3	4
There are people I feel close to.	1	2	3	4
I feel left out.	1	2	3	4
My social relationships are superficial.	1	2	3	4
No one really knows me well.	1	2	3	4
I feel isolated from others.	1	2	3	4
I can find companionship when I want it.	1	2	3	4
There are people who really understand me.	1	2	3	4
I am unhappy being so withdrawn.	1	2	3	4
People are around me but not with me.	1	2	3	4
There are people I can talk to.	1	2	3	4
There are people I can turn to.	1	2	3	4

Japanese version.

	1: 決して感じない	2: どちらかと言えば感じない	3: どちらかと言えば感じる	4: たびたび感じる
私は、自分の周囲の人たちと調子ょくいっている	1	2	3	4
私は、人とのつきあいがない	1	2	3	4
私には、頼りにできる人がだれもいない	1	2	3	4
私は、ひとりぼっちではない	1	2	3	4
私は、親しい仲間たちのなかで欠くことのできない存在である	1	2	3	4
私は、自分の周囲の人たちと共通点が多い	1	2	3	4
私は、今、だれとも親しくしていない	1	2	3	4
私の興味や考えは、私の周囲の人たちとはちがう	1	2	3	4
私は、外出好きの人間である	1	2	3	4
私には、親密感のもてる人たちがいる	1	2	3	4
私は、無視されている	1	2	3	4
私の社会的なつながりはうわべだけのものである	1	2	3	4
私をよく知っている人はだれもいない	1	2	3	4
私は、他の人たちから孤立している	1	2	3	4

私は、望むときにはいつでも、人とつきあうことができる	1	2	3	4
私には、私を本当に理解してくれる人たちがいる	1	2	3	4
私は、たいへん引っ込み思案なのでみじめである	1	2	3	4
私には、知人はいるが、私と同じ考えの人はいない	1	2	3	4
私には、話しかけることのできる人たちがいる	1	2	3	4
私には、頼りにできる人たちがいる	1	2	3	4
