# Supplementary material for: Cultural differences in social support seeking: The mediating role of empathic concern
Source: PLoS One. 2021 Dec 30;16(12):e0262001. doi: 10.1371/journal.pone.0262001 (PMC8718000; doi:10.1371/journal.pone.0262001)
Supplement: S1 Table — (PDF) [file pone.0262001.s002.pdf]

S1 Table. Results of emotional support seeking and instrumental support seeking.

	Indirect effect	*SE*	95% CI
Study1			
culture → empathic concern → emotional support seeking	0.126	0.027	[0.079, 0.189]
culture → empathic concern → instrumental support seeking	0.108	0.026	[0.066, 0.168]
Study 2			
culture → empathic concern → emotional support seeking → loneliness	-0.016	0.004	[-0.025, -0.009]
culture → relational concern → emotional support seeking → loneliness	-0.005	0.002	[-0.009, -0.002]
culture → empathic concern → instrumental support seeking → loneliness	-0.012	0.004	[-0.020, -0.006]
culture → relational concern → instrumental support seeking → loneliness	-0.006	0.002	[-0.011, -0.002]

Note. In Study 1, age and gender were included as control variables, whereas in Study 2, besides gender and age, SES and 5 related feelings for the stressful events were also included as control variables.
