# Supplementary material for: Cultural differences in social support seeking: The mediating role of empathic concern
Source: PLoS One. 2021 Dec 30;16(12):e0262001. doi: 10.1371/journal.pone.0262001 (PMC8718000; doi:10.1371/journal.pone.0262001)
Supplement: S2 Table — (PDF) [file pone.0262001.s003.pdf]

S2 Table. Means of support seeking and loneliness by COVID-19 in Study 2.

		None		COVID-19					
		Mean	*SD*	Mean	*SD*	*t*	*df*	*p*	Cohen's *d*
Japan		(*N* = 383)		(*N* = 112)					
	Support seeking	2.53	0.95	2.52	0.88	0.04	493	.970	0.004
	Loneliness	2.47	0.62	2.38	0.60	1.32	493	.188	0.141
U.S.		(*N* = 273)		(*N* = 188)					
	Support seeking	2.91	1.08	3.01	0.95	-1.08	432.10	.282	-0.100
	Loneliness	2.08	0.66	1.97	0.61	1.75	459	.081	0.166

The potential influence of COVID-19

The data of Study 2 were collected during the outbreak of COVID-19. According to whether participants mentioned COVID-19 in their description of the stressful event, we categorized participants into two groups (None vs. COVID-19) to examine whether COVID-19 would influence participants' support seeking behaviors and loneliness.

The results of Independent T-tests showed that although European American (40.8%) mentioned COVID-19 more than Japanese (22.6%), mentioning COVID-19 did not impact support seeking and loneliness among both samples (*p*s > .08; S1 Table). Therefore, we did not include COVID-19 in the main analyses.
