# Supplementary material for: Cultural differences in social support seeking: The mediating role of empathic concern
Source: PLoS One. 2021 Dec 30;16(12):e0262001. doi: 10.1371/journal.pone.0262001 (PMC8718000; doi:10.1371/journal.pone.0262001)
Supplement: S1 Text — (PDF) [file pone.0262001.s004.pdf]

S1 Text. Results using the whole scale of relational concern (13 items).

The scale of relational concern that previous studies used were 13 items including two factors: relational concern about seeking social support actively (11 items) and expectation of unsolicited social support (2 items) (e.g., Kim et al., 2006). Our research only focused on relational concern. Therefore, we excluded the items assessing the expectation of unsolicited social support in the main analyses. Here, we presented the results of using all 13 items.

First, the internal consistency of the full scale (13 items) was good. Cronbach's alphas were 0.83 for the Japanese and 0.91 for the European Americans. The results of Independent T-tests showed that compared to the Japanese participants ($M = 2.86$, $SD = 0.62$), European American participants ($M = 2.45$, $SD = 0.84$) reported less relational concern ($t(844) = 8.35$, $p < .001$, Cohen's $d = 0.55$). And relational concern correlated with support seeking negatively (Japan: $r = -0.09$, $p = .050$; America: $r = -0.17$, $p < .001$).

As for the results of Mediation analyses (Bootstrapping $N = 10{,}000$), both empathic concern and relational concern mediated the cultural differences in support seeking significantly: Indirect effect = 0.12, $SE = 0.03$, 95% CI = [0.07, 0.17] for empathic concern; Indirect effect = 0.04, $SE = 0.02$, 95% CI = [0.02, 0.08] for relational concern. And the indirect effect of empathic concern was stronger than that of relational concern $d = 0.08$, $SE = 0.03$, 95% CI = [0.02, 0.14]. Furthermore, the serial mediating effects were also supported: Indirect effect = -0.02, $SE = 0.00$, 95% CI = [-0.03, -0.01] for culture → empathic concern → support seeking → loneliness; Indirect effect = -0.01, $SE = 0.00$, 95% CI = [-0.01, -0.00] for culture → relational concern → support seeking → loneliness. Besides, the indirect effect of culture differences in loneliness through support seeking was also significant, Indirect effect = -0.03, $SE = 0.01$, 95% CI = [-0.05, -0.01].
