# Supplementary material for: Cultural differences in social support seeking: The mediating role of empathic concern
Source: PLoS One. 2021 Dec 30;16(12):e0262001. doi: 10.1371/journal.pone.0262001 (PMC8718000; doi:10.1371/journal.pone.0262001)
Supplement: Attachment — Submitted filename: Social support seeking & empathic concern.pdf [file pone.0262001.s008.pdf]

This revision is responsive to many of the issues raised in the review of the original submission, and hence is much improved. However, the two most fundamental issues are not resolved. Since both of these issues are common to most research in cultural psychology, perhaps the best course of action is to acknowledge that them frankly from the beginning, and simply publish the paper. But I must say that what I see as the fundamental validity problems of most research in cultural psychology are salient in this paper.

The issue of cross-national comparison of Likert scale values *is* frankly, though all too briefly, addressed in the next to last paragraph, though I suggest that the authors add a few sentences about better methods.

However, the authors continue to rely on the individualism – collectivism dichotomy, despite all the evidence that these are invalid constructs, that there is too much heterogeneity within nations to allow inferences as the national level, that different measures of the do not agree, that measures of them find dimensions rather than distinct categories, and that most studies that measure both constructs find little correlation between them (so they are not opposite ends of one dimension). Why do the authors base their metatheory on these invalidated constructs? My opinion is that they should at least acknowledge the many respects in which the constructs are problematic. However, I can also accept an editorial decision that doing so is beyond the scope of this paper. Even if this is the decision, however, there are component problems that I favor addressing.

Could the starting point for the whole paper be wholly or partially a methodological artifact? The authors indicate that East Asian cultures (all? some? there are a great many East Asian cultures) are characterized by the judgment that presenting distress to another person typically(?) is an unwarranted or at least unwelcome imposition. If so, then one would expect social desirability effects such that survey respondents would under-report support-seeking. This likelihood needs to be carefully addressed.

Let me return to the issue of comparing distributions of responses on items in different languages. In the wording of a question, even small differences *within* a language may yield large quantitative differences in responses. But here we are comparing responses to items in different languages. The differences in social support-seeking in these studies and those reported in previous studies are based on comparing scales anchored by English words such as *rarely, very much,* and so forth, with scales in Mandarin, Cantonese, Japanese, Malay, Vietnamese, Thai, or Korean lexemes with similarly indeterminate meanings that surely do not align perfectly with the English frequency terms anchoring the scales for the North Americans. Back-translation is a start, but what one inevitably discovers doing back translation is that it is just not possible to "ensure cross-cultural equivalence" of the purportedly corresponding items in two languages. Hence differences between nations in the distributions of responses cannot be used to make valid inferences about the difference in the absolute distribution of responses in the samples (including their means).

Furthermore, the lexemes used to describe forms of "social support-seeking", or the term for "stress", or the term "solvable" or "responsible", or the lexeme for "close to" in a scale in English cannot possibly have denotations (nor connotative valuation) identical to the lexemes used to describe those constructs in any other language – here, Japanese. Languages simply do not have lexicons that that construe the

world according to any universal ontology or taxonomy: lexicons do not align in one-to-one correspondence.

In addition, in Study 2 the authors found substantial differences in the problems for which US respondents potentially sought social support, compared to the Japanese respondents; in particular, the Japanese respondents reported more negative and more stressful events (though one does not know whether these are true differences, or result from differences in the meanings of the scales in the respective languages). Given that finding, how meaningful is it to compare the social support-seeking of the two samples? Surely the nature of the problems one faces has a large effect on whether one seeks support, what support one seeks, and from whom.

Beyond that, the words, gestures, facial expressions and other actions that comprise "support-seeking" in Japan are doubtless different from those that are typical in Canada, or in the US. That is, the very acts of support-seeking differ between the two nations, meaning that the studies compare apples in North America to oranges in Japan. Furthermore, the "support" that is sought surely differs across cultures, nations, and regions. For example, Adams & Plaut (2003) found that, in contrast to European Americans, West Africans expect financial support from friends, but rarely seek advice and even less often expect sympathetic listening to expressed emotions. (My own informal observations in the course of over 4 years in West Africa support this.) So, in sum, just what were the Japanese respondents reporting that they were seeking, and how were they seeking it? What were the Canadian and what were the US respondents seeking (not necessarily the same thing, as the two nations contain somewhat different sets of cultures), and what did seeking consist of?

All in all, I believe that the authors need to assiduously unpack and discuss the meanings of the prompts and of the scale anchors in the respective languages. A Likert self-report scale is not a caliper that can be reliably calibrated so as to yield objective and truly comparable measurements, and Likert self-report scales in (very) different languages simply do not measure the exact same behaviors, let alone absolute frequency distributions of behaviors in any of the samples. In short, can one truly conclude that there are, in fact, between-nation differences in the rates of behaviors related to the construct of "social support-seeking" – especially if people in the respective nations are not seeking exactly the same sort of support in the same way?

In any case, I do not have great confidence in the validity of claims such as, "compared to Americans, Japanese and Chinese individuals reported greater degrees of loneliness". And if Japanese are more lonely, how does that comport with the notion that Japanese culture is more 'collectivist' than North America? Likewise, I doubt the validity of the evidence that, "compared to East Asians, Westerners are more likely to empathize with people in distress by exhibiting sympathy (e.g., [27]). For instance, Cassels et al. [28] found that East Asian adolescents reported lower empathic concern than did Western adolescents, whereas mainland Chinese university students scored lower in empathic concern assessments than German undergraduates [29, 30]. Moreover, American counselor trainees showed greater empathic concern than their Thai counterparts [31] . . . . East Asians show less empathic concern than do Westerners." Simply running Student's t-tests and ANOVAs on Likert scales in two different languages does not constitute evidence for such conclusions. The authors have added a short penultimate paragraph to acknowledge this, but then, why do they make the inferences they do make throughout the paper?

Again, let me say that I realize that this critique applies to most research in the cross-cultural psychology paradigm. Yes.

These issues are probably somewhat less vitiating for mediational analyses (so long as one does not *compare the magnitudes* of mediations in samples studied with instruments in different languages). Hence there remains a lot of value in these studies. I would suggest that the authors could greatly strengthen the interesting but unsupported inference that people (in general) are less disposed to seek social support when they view support-seeking as an imposition on relationships, and hence potentially deleterious to them. To explore this, why not *ask* informants if this is indeed what they think? It would be very illuminating to do 20-30 interviews and a couple of focus groups in Japan, and the same in North America. My intuition is that, for the most part, North American recipients of requests for social support feel that such requests are flattering, even moving, indicators of trust and closeness. I believe that in my own North American subculture, support-seeking strengthens friendships and family bonds.

What is the evidence that East Asians "are less likely to experience emotion more intensively"? That claim astonishes me. And how does this mesh, for example, with the fact that suicide rates in Japan are nearly twice as high as in, say, the United Kingdom? Or with, say, the Japanese preoccupation with *kawaii*? Suicide and *kawaii*-related practices manifest themselves quite publicly, so how does this comport with the claim that East Asians are less likely to "express their emotion publicly"? At baseball games?

How were non-European participants excluded from the Alberta sample – and what definition of "European Canadian" was used to do so? Likewise for study 2, how were non-European Americans defined and how were they excluded?

In the general discussion, the authors write that "individuals from Japan display less empathic concern for people in distress. These cultural differences result from the cultural differences in emotional expressivity and cognitive thinking styles. On the one hand, East Asians, with their strong motivation to maintain interpersonal harmony, compared to Westerners, are more cautious about their emotional responses in interpersonal contexts (e.g., [10]). The high tendency to restrain and suppress emotion in daily life may hinder East Asians from empathizing with unfortunate others". As far as I can see, their studies do not address the claims that I underline, nor do the authors offer any other evidence for these causal inferences. (And what does "more cautious" mean?) Is this claim about all emotions?

The authors note that "compared to Americans, Chinese people are more likely to perceive unfortunates as responsible for and deserving of their misfortune, and they reported less overall sympathy [41]. In Study 2, we also consistently found that, compared to European Americans, Japanese people reported more responsibilities for their own stressors". To me these results seem completely at odds with the characterization of Chinese or Japanese as 'collectivist' and European Americans as 'individualist'. Individualists should believe that people are individually responsible for what happens to them, while collectivists should believe that misfortunes and stressors are due to the overall circumstances, roles and relationships, not the individual, personally. Indeed, Joan Miller found precisely this contrast between explanations for real events offered by her samples in Chicago and Orissa, India (sorry, I can't recall the reference – from the 1970's, I believe). I find that I don't understand what construct of 'collectivism' the authors are using.

It seems to me that “turning to others in times of need helps restore one’s sense of belonging” would be especially true, or at least especially prevalent, among ‘collectivists. In any case, if turning to others in times of need helps restore one’s sense of “belonging,” how does belonging differ from a “relationship”? Why does such support-seeking strengthen belonging but threaten relationships?

I reiterate that the authors of this paper operate in a paradigm that I find problematic – I have essentially the same problems with nearly all research in cultural psychology. So an editorial decision to put aside my critique would be reasonable. On the other hand, paradigms only change when they are challenged study by study, method by method, findings by findings.

Reference:

Adams, G., & Plaut, V. C. (2003). The cultural grounding of personal relationship: Friendship in North American and West African worlds. *Personal Relationships*, *10*(3), 333-347.
