# Supplementary material for: Cultural differences in social support seeking: The mediating role of empathic concern
Source: PLoS One. 2021 Dec 30;16(12):e0262001. doi: 10.1371/journal.pone.0262001 (PMC8718000; doi:10.1371/journal.pone.0262001)
Supplement: Attachment — Submitted filename: Response to Reviewers.doc [file pone.0262001.s007.doc]

Professor Frantisek Sudzina

Academic Editor, *PLoS ONE*

Aalborg University

Dear Professor Sudzina,

We have completed a revision of our paper entitled “Cultural Differences in Social Support Seeking: The mediating Role of Empathic Concern” (PONE-D-20-40124). We are very grateful for the time and effort you and your reviewer generously expended on this paper. In revising this paper, the reviewer’s comments were very helpful. In what follows, let me explain the changes (indicated in red in the revised manuscript) we have made on the paper to reply to the reviewers’ comments.

Journal Requirements

1. *Please ensure that your manuscript meets PLOS ONE's style requirements, including those for file naming.*

Following the instruction on the journal webpage, we reformatted our manuscript.

2. *Please change "female" or "male" to "woman" or "man" as appropriate,
   when used as a noun.*

Following this suggestion, we revised the participants section of each study (lines 200-203 on page 10 and lines 299-300 on page 14) and Table 2.

3. *We note you have included a table to which you do not refer in the text of your manuscript. Please ensure that you refer to Table 4 in your text.*

This revised manuscript does not either include Table 4 or mention it in the main text.

4. *Please include captions for your Supporting Information files at the end of your manuscript, and update any in-text citations to match accordingly.*

Following this suggestion, we included captions for our supporting information files at the end of our manuscript and updated in-text citations.

Reviewer #1

*However, I recommend that the paper be rewritten. First, while the authors' summary of the health effects of loneliness vs. social support are valid, their sources are out of date and exclude a number of important meta-analyses. That is easy to fix; I list three important sources below, but there are others as well.*

Thank you for your suggestion. We added some new reviews/ meta-analyses of the health effects of social support and loneliness (Barth et al. [2010]; Chida et al., [2008]; Chu et al., [2010]; Holt-Lunstad et al., [2015]), which include ones you suggested (see [2], [3], [4], and [68] in the main text).

*Second, like many others, the authors frame their conceptualization and design in terms of the constructs of individualism and collectivism. Research shows that these are at best orthogonal dimensions, and certainly not dichotomous discrete categories. Indeed, meta-analyses strongly indicate that neither construct is valid. For one critique, see Fiske (2002) and the other comments in that issue.*

Previous research of culture and social support assumes the negative implications of social support seeking for interpersonal relationships, such as bothering others and losing face, in cultures (or nations) emphasizing social networks and accommodation to others. Particularly, disclosing problems and bringing personal matters to others are relatively inappropriate in Eastern cultures, because such a disclosure implies a demand for help from others, which may damage a harmonious relationship. This assumption is thus based on the norm of interpersonal relationships reflecting the interdependent orientation. On the other hand, according to Miller, Akiyama, and Kapadia (2017, *Journal of Personality and Social Psychology*), hesitation in seeking social support, reflecting relational concern on disrupting harmonious relationships, seems to be inconsistent with communal norms that have been considered as a core feature of collectivism (Oyserman et al., 2002). Miller et al. (2017) thus call into question two claims: one is that collectivism is associated with hesitation in seeking social support, and the other one is that the feature of hesitation in seeking social support, reflecting the interdependent orientation, is generalized in Asians (indeed, they found Indians who likely to engage in communal norms less hesitate social support seeking compared to Japanese). Given your comment and the findings of Miller et al. (2017), we reframed the introduction of our manuscript along with the previous research on culture and social support, not mentioning the dimension of individualism-collectivism (on pages 3-5). Additionally, based on Miller et al. (2017), we mentioned a possibility that the association between relational concern and hesitation in social support seeking may be applied to a limited group of East Asians on page 28.

*Third, any time one reports studies using translations of scales, one needs to report the precise items in each language, then discuss and carefully consider the implications for data analyses of the differences in meanings of the items in the respective languages.*

Based on this comment, we included the English and Japanese versions of all the scales we used in Supporting Information (S1 Appendix) to make them public so that people who are interested in the scales can evaluate the validity. Also, we added some words to support cross-cultural validity for each scale (on pages 11, 16 and 17). Furthermore, we mentioned an issue of culturally biased item wordings by referring tot Fiske (2002) on page 28.

*Finally while many researchers infer differences between nations (or cultures) by comparing means on Likert scales, such inferences are invalid. There are several reasons for this invalidity, including the fact that one cannot meaningfully compare the means of items written in different languages. Respondents in different cultures are also likely to anchor their responses differently - e.g., basing their responses on comparison of their beliefs about themselves with their beliefs about others in their own culture. Making meaningful comparisons between nations (or cultures) requires systematic ethnological research based on analyses of ethnographies, as well as consultation with social scientists who study both of the two cultures. At a bare minimum, one needs to use scenario items (Peng, Nisbett & Wong 1997; Heine, Lehman, Peng, & Greenholtz 2002). In short, I recommend that the authors not use previous studies or their own data to infer mean international differences in any of their variables.*

As you pointed out, we admit that the problem of mean-level cross-cultural comparison using Likert scales is one of the limitations of this research. We mentioned the problem by referring to Heine et al. (2002) and Peng et al. (1997) on page 28. However, please note that our hypothesis is not based on cultural differences in traits and thoughts themselves people support. Rather, we focus on how a set of variables related to relationships and emotional experiences are functionally related and whether culture moderates the functional relationships. Following Kitayama (2002), which is one of the comments to Oyserman et al. (2002), we take a stand for the system view of culture.

*So I strongly urge the authors to reframe their conceptualization and analyses as replications in three nations showing that social support seeking is affected by relational concerns, mediated by empathic concern and "social norms." And I encourage a deeper conceptualization of "social norms" and how they operate in this case. This would make a solid and valuable article.*

In this revision, reframing the introduction of culture and social support seeking by addressing the assumption of the previous research, we emphasized the roles of relational concern and empathic concern as mediators. For example, we mentioned that the results (i.e., compared to European Americans, Japanese individuals with higher levels of relational concern less frequently sought social support during stressful times) replicated the previous findings on page 24 (see also page 25). Moreover, given that independence and interdependence are multifaceted, we clarified our argument that to see a possibility that other factors related to culturally sanctioned ways of self and relationships can also contribute to cultural differences in social support seeking, we explore the role of empathic concern, which differs reflecting cultural norms regarding emotional expressivity and cognitive styles (pages 5-7). Furthermore, we referred to Miller et al. (2017) to address a possibility that norms about relationships reflecting an interdependent view of self and emphasis on either communal norms or exchange norms interact to affect hesitation and discomfort in social support seeking (page 28), because we think that future work addressing it is crucial to gain more integrated understanding about the cultural underpinnings of using social support. Finally, we also referred to potential effects of regional variations and socio-ecological variations, which can lead to differences in the extent to which individuals adhere to the dominant value of interdependence on page 28.

Reviewer #2 *The framing of the paper could use improvement. First, it is important to be specific about the cultures being investigated. The literature review moves back and forth between different conceptualizations, the connections among which are not clear. For example, are the phenomena in question specific to Japan? To all East Asian countries? Do they also include those in other countries of East Asian descent, such as Chinese
Americans? Similarly, the comparison groups vary widely, including Canadians of European descent, Americans of British descent, etc. (The latter is not the majority of U.S. citizens, so this comparison is not particularly relevant to understanding country level differences in help-seeking.) Care should also be taken not to use "Canadians" or
"Americans" when what is meant is a subgroup of the population.*

Following this suggestion, we revised the framing of the introduction section to focus on East Asian culture and Western culture. And we also added some sentences to make the connection between different conceptualization clearer. Second, the reviewer pointed out that European Americans cannot represent “Americans.” Following this, we replaced the terms of “Canadians” and “Americans” with “European Canadians” and “European Americans” respectively.

*The underlying arguments regarding culture and empathic concern are quite interesting, and as the authors note, some of the prior findings would appear on the surface to be contradictory. However, the line of argument is likely to be hard to follow without a more general introduction to the cultural attributes of the cultures under comparison (e.g., individualism/collectivism, self-concept, etc.). This could make subsequent discussion of possible reasons for cultural differences in help seeking easier to follow, particularly for readers who do not work in this area of research.*

You pointed out that the arguments regarding cultural variations in empathic concern may be hard to follow without a more general introduction to the cultural attributes of the cultures under comparison. Following this suggestion, we added some sentences (line 55-64 on page 3-4) to introduce the distinct attributes of social relationship in each culture to help understand the cultural variations in relational concern. And we also described the attributes of each culture in emotional expressivity and cognitive thinking styles briefly at the beginning of 8^th^ (line 122-125 on page 6) and 9^th^ paragraph (line 131-134 page 7).

*The paper would also be easier to follow if all key terms and phrases were defined on first use, including "empathic concern" in the first paragraph and abstract; "implicit social support" in paragraph 2, and so on.*

Following this suggestion, we added the definition of empathic concern in the abstract (the 3^rd^ sentence, line 23 on page 2). We deleted “implicit social support” in this revised manuscript. And the definition of social support was moved to the first sentence in the first paragraph (line 37-38 on page 3). The definition of loneliness is on line 154-155 (page 8).

*My main concern is that comparing surveys distributed to different cultural groups is tricky and the authors do not seem to have considered cultural differences in the ways in which people respond to subjective scales. There is a large literature showing that simply translating a scale between languages does not ensure that the respondents are conceptualizing the different values on the scale in the same way. There are also related issues pertaining to social desirability in responses, which also varies across cultures.*

As you pointed out, researchers have proposed several methods to improve the comparability of cross-cultural questionnaires. However, those statistical methods cannot effectively solve the major problems of cross-cultural comparison on Likert scales that Reviewer 1 mentioned (i.e., the reference group effect). There is only one way to address this issue that is to examine these findings with multiple methods in future. Therefore, combined with Reviewer 1’s last comment, we extended our argument on page 28.

We hope that the present version is much clearer. We cannot thank you more for your advice and guidance. Please feel free to contact us if you have any questions or need further materials.

Sincerely,

Shaofeng Zheng and Keiko Ishii (on behalf of the authors)
